# Synthesis and detection the oxidization of Co cores of Co@SiO_2_ core-shell nanoparticles by *in situ* XRD and EXAFS

**DOI:** 10.1186/s11671-015-0756-z

**Published:** 2015-02-05

**Authors:** Kunhao Zhang, Ziyan Zhao, Zhonghua Wu, Ying Zhou

**Affiliations:** Department of Life Science, Shanghai Synchrotron Radiation Facility, Shanghai Institute of Applied Physics, Chinese Academy of Sciences, Shanghai, 201204 China; State Key Laboratory of Oil and Gas Reservoir and Exploitation, Southwest Petroleum University, Chengdu, 610500 China; School of Materials Science and Engineering, Southwest Petroleum University, Chengdu, 610500 China; Beijing Synchrotron Radiation Facility, Institute of High Energy Physics, Chinese Academy of Sciences, Beijing, 100049 China

**Keywords:** Core-shell nanoparticles, Co@SiO_2_, *In situ* XRD/XAFS, Synchrotron radiation techniques

## Abstract

In this paper, the Co@SiO_2_ core-shell nanoparticles were prepared by the sol-gel method. The oxidization of Co core nanoparticles was studied by the synchrotron radiation-based techniques including *in situ* X-ray diffraction (XRD) and X-ray absorption fine structure (XAFS) up to 800°C in air and N_2_ protection conditions, respectively. It was found that the oxidization of Co cores is undergoing three steps regardless of being in air or in N_2_ protection condition. In the first step ranging from room temperature to 200°C, the Co cores were dominated by Co^0^ state as well as small amount of Co^2+^ ions. When temperature was above 300°C, the interface between Co cores and SiO_2_ shells was gradually oxidized into Co^2+^, and the CoO layer was observed. As the temperature increasing to 800°C, the Co cores were oxidized to Co_3_O_4_ or Co_3_O_4_/CoO. Nevertheless, the oxidization kinetics of Co cores is different for the Co@SiO_2_ in air and N_2_ gas conditions. Generally, the O_2_ in the air could get through the SiO_2_ shells easily onto the Co core surface and induce the oxidization of the Co cores due to the mesoporous nature of the SiO_2_ shells. However, in N_2_ gas condition, the O atoms can only be from the SiO_2_ shells, so the diffusion effect of O atoms in the interface between Co core and SiO_2_ shell plays a key role.

## Background

In the past years, nanomaterials have been attracted extensive interests due to their unique properties and potential applications in chemistry, physics, biology, and catalysis. For example, magnetic nanoparticles have potential applications in catalyst, resonance imaging, drug targeting, and bio-conjugation. However, the magnetic nanoparticles can be oxidized easily in atmosphere and thus limiting the applications of these nanomaterials [1-3].

Recently, a series of supported cobalt or cobalt oxide materials such as Co/Al_2_O_3_, Co/κ-Al_2_O_3_, Co/SiO_2_, and Co/TiO_2_ have been studied for catalysis. The most famous application of the Co/SiO_2_ and Co/Al_2_O_3_ catalysts is for the Fischer-Tropsch synthesis [4-8]. W. Ma and T. Das investigated the influence of support type and cobalt cluster size on the kinetics of Fischer-Tropsch synthesis of Co/SiO_2_ catalysts, and the kinetic results demonstrated that the Fischer-Tropsch reaction exhibited some structure sensitivity to the kinetic effect of water with respect to support type and Co cluster size [5,6]. A. M. Saib studied the surface oxidation behavior of the nanosized cobalt crystallites (4 to 5 nm) of Co/SiO_2_/Si(100) model catalyst using *in situ* near-edge X-ray absorption fine structure (NEXAFS) under model Fischer-Tropsch synthesis conditions. No surface oxidation of cobalt was observed under these model FTS conditions over a wide temperature range, i.e., 150°C to 400°C [7]. The Co/SiO_2_ materials can be used as catalyst for hydrogen generation as well [9]. In general, it has been reported that the Co_3_O_4_ particles were more readily reduced to metallic cobalt in H_2_ than the Co^2+^ species. After reduction at 480°C in H_2_, the CO hydrogenation activity in ten atmospheres of 3H_2_:1CO at 260°C with supported 5 wt% cobalt decreased as the order of Co/SiO_2_ > Co/TiO_2_ > Co/Al_2_O_3_ > Co/κ-Al_2_O_3_. Therefore, the determination of the types of cobalt species present on each support and their reduction properties was to the key points to explain the catalysts' CO hydrogenation activities [10].

Different strategies have been proposed for the preparation of Co/oxide core-shell nanoparticles. X. J. Yin and X. Lu have synthesized the Co/SiO_2_ core-shell nanoparticles using the novel aqueous solution method and improved sol-gel method combining with hydrogen reduction, and they also found that the saturation magnetization and coercivity varies with the SiO_2_ content. The size and the saturation magnetization value of samples decreased with the increase of the SiO_2_ content [11,12]. In order to protect the oxidation of magnetic nanoparticles, an inert shell onto the magnetic core nanoparticles could be an elegant approach. V. Salgueiriño-Maceira et al. reported a sol-gel method to synthesize the Co nanoparticles which are coated with a protective silica layer and then using the standard Stŏber (by adding the tetraethoxysilane (TEOS) into aqueous/ethanolic solution) method to obtain the Co@SiO_2_ core-shell nanoparticles. They have also reported the first synthesis of unique silica-coated chains of 32-nm cobalt nanoparticles resembling nanoscale pearl necklaces in colloidal suspension under magnetic stirring. This phenomenon was attributed to the magnetic dipole-dipole interaction between neighbor particles [13,14]. Up to now, there are many magnetic core-shell materials which have been made including Fe_2_O_3_@SiO_2_/Ag, Fe_3_O_4_@SiO_2_, Fe_3_O_4_@SnO_2_, Co@SiO_2_, Pt@CoO, FePt@SiO_2_, Fe_3_O_4_@Au, Fe_2_O_3_-CdSe@SiO_2_, and Fe_3_O_4_/γ-Fe_2_O_3_@SiO_2_ [15-23]. For example, the Fe_3_O_4_@SiO_2_ is a common magnetic core-shell nanoparticle. The core particle Fe_3_O_4_ can be used in resonance imaging, whereas the shell layer is mesoporous SiO_2_, which can provide enough space for additive and can be used for loading particles to adsorb or isolate protein and antibody. Moreover, through the surface modification of the shell layer by adsorbing noble metal nanoparticles, the core-shell system can be used for catalyst, luminescence imaging, and photodynamic therapy [24].

However, the stability and thermal properties of Co@SiO_2_ under high temperature have not been completely studied. In this paper, the *in situ* extended X-ray absorption fine structure (EXAFS) and X-ray diffraction (XRD) techniques are used to probe the properties of Co@SiO_2_ core-shell nanoparticles with temperature up to 800°C.

## Methods

### Chemical reagents

Cobalt chloride hexahydrate (CoCl_2_ · 6H_2_O), sodium borohydride (NaBH_4_), sodium citrate dehydrate, and anhydrous ethanol were purchased from Sinopharm Chemical Reagent Beijing Co., Ltd., China. TEOS and 3-aminopropyltriethoxysilane (APS) were purchased from Sigma-Aldrich, St. Louis, MO, USA. All reagents were used as received. Deionized water was distilled by a Milli-Q water purification system (Millipore Corp., Bedford, MA, USA).

### Preparation of Co@SiO_2_ core-shell nanoparticles

Co@SiO_2_ core-shell nanoparticles were prepared by V. Salgueiriño-Maceira's method [13,14]. Firstly, citrate stabilized Co nanoparticles were prepared from the conventional NaBH_4_ reduction of CoCl_2_ · 6H_2_O. In a typical procedure, under vigorous stirring and N_2_ protection, 0.2 mL of 0.4 M CoCl_2_ solution was added quickly into 200 mL water which contains 4 × 10^−3^ M NaBH_4_ and 4 × 10^−4^ M sodium citrate. The solution turned brown or black immediately after mixing. Secondly, 800 mL ethanol with 14.4 μL APS and 169 μL TEOS was added into the above solution after 1 min and then kept stirring at least 24 h to complete the reaction. Finally, the Co@SiO_2_ core-shell nanoparticles were separated by centrifugation and dried in air for further investigation.

### Transmission electron microscopy

Bright-field transmission electron microscopy (TEM) observation was performed on a JEM 1230 electron microscope (JEOL Ltd., Akishima-shi, Japan) operated at 80 kV. The specimens were prepared by dropping the Co@SiO_2_ solution onto a carbon-coated TEM grid. After the specimens were dried in air, they were used for the TEM observation.

### Ultraviolet-visible absorption spectroscopy

During the preparation of Co@SiO_2_ nanoparticles, the color of the solution changing from colorless to brown was observed, indicating that the Co^2+^ ions have been reduced to Co nanoparticles. Moreover, in the period of silica-coating procedure, the surround mediate of Co nanoparticles changed which could inflect the absorption cross section. So we used the Nicolet Evolution 300 spectrophotometer (Thermal Fisher Scientific, Waltham, MA, USA) to invest the ultraviolet-visible (UV-vis) absorption spectroscopy of the reaction solution. The wavelength range is 190 ~ 1,100 nm.

### Extended X-ray absorption fine structure measurements

Transmission EXAFS measurements of Co K edge (7,709 eV) were performed at the beamline 4B9A of Beijing Synchrotron Radiation Facility (BSRF). The storage ring was operated at 2.5 GeV with current about 200 mA. The EXAFS signals in the energy range from 7,589 to 8,709 eV were collected with two ionization chambers filled with 100% N_2_ gas. The incident X-ray was monochromatized with a double-crystal Si (111) monochromator to an energy resolution (*ΔE*/*E*) of 2 × 10^−4^. In order to take *in situ* EXAFS measurements, the Co@SiO_2_ should mix with BN powder and was pressed into a pill of 10 mm in diameter and 1 mm in thickness (*d*). By adjusting the ratio of Co@SiO_2_ and BN in the mixture, the absorption thickness (*Δμ* · *d*) was optimized to one, where *Δμ* is the difference of Co absorption coefficients after and before the Co K absorption edge (7,709 eV). Then, the pill was placed on the sample holder which can be inserted into the heating furnace. The temperature uncertainty can be controlled within ±0.1°C with an 818 temperature controller. During heating the sample, the heating rate was set to 10°C/min. The room temperature EXAFS spectrum was first collected, and subsequently, the high-temperature EXAFS spectra were orderly collected in the temperature range from 100°C to 800°C with a temperature interval of 100°C. Before EXAFS measurements at each target temperature, the sample was heat preserved at least 30 min to ensure the sample reaching a thermal equilibrium. In order to invest the influence of reaction atmosphere's to Co oxidation process, we made the EXAFS measurements under air and N_2_ conditions.

### X-ray diffraction measurements

*In situ* XRD of the Co@SiO_2_ core-shell nanoparticles was measured at the beamline 4B9A-XRD of BSRF using an image plate. The diffraction signals were collected after the EXAFS measurements at each target temperature. As same as the EXAFS, the temperature range is 25°C ~ 800°C.

## Results and discussion

**Figure 1:**
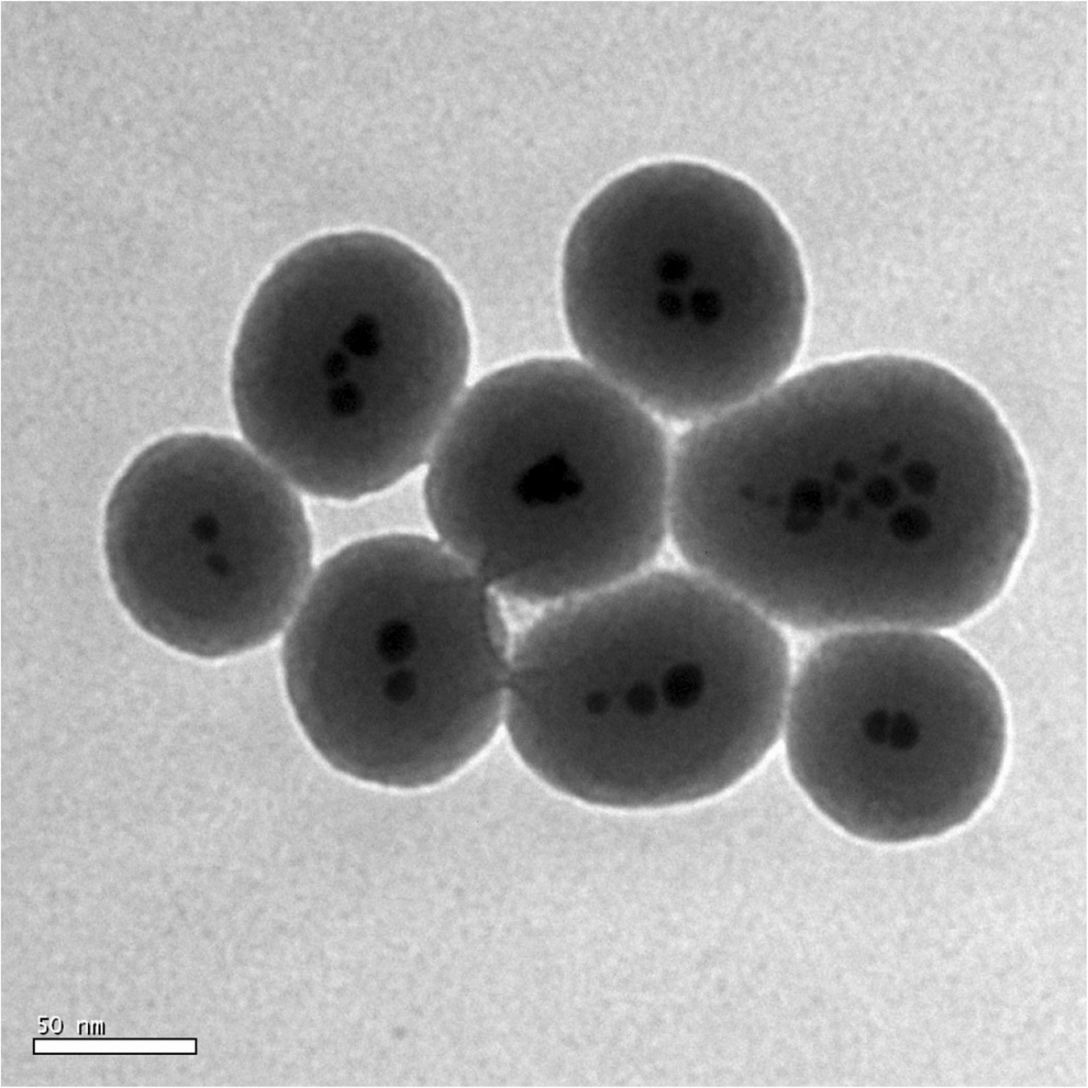
**TEM image of the as-prepared Co@SiO**_**2**_ **core-shell nanoparticles.**

The TEM image of the Co@SiO_2_ core-shell nanoparticles is shown in Figure 1. Most of the Co@SiO_2_ nanoparticles with ~50 nm diameter contain multiple Co cores, but the Co cores are separated from each other. According to the TEM image, the average diameter of Co cores is evaluated to be about 20 nm. The obtained Co@SiO_2_ core-shell nanoparticles are different from the previous work [13,14] which may be due to the different reaction conditions, such as the rate of protect N_2_ gas and stirring rate.

**Figure 2:**
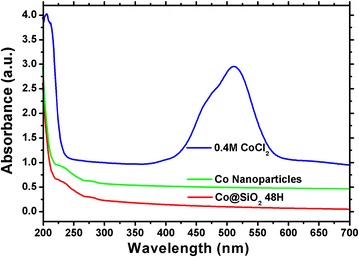
**UV-vis spectra of solution during synthesis time.**

Figure 2 shows the UV-vis spectroscopy during the reaction process. The initial CoCl_2_ solution exhibits a high absorption peak at 510 nm (blue line), which is disappeared immediately after the addition of NaBH_4_ solution. In the meantime, there are two weak absorption peaks at 230 and 280 nm which belong to the Co nanoparticles (yellow line). Based on these results, it reveals that the Co nanoparticles are synthesized immediately after the addition of NaBH_4_ solution. The UV-vis spectroscopy of Co@SiO_2_ core-shell nanoparticles after the addition of APS and TEOS (red line) was measured as well (cf. Figure 2). No significant change from the Co nanoparticles was observed, except the higher intensities of the absorption peaks. This is because that the SiO_2_ shell could change the dielectric constant around Co cores and thus increases the absorption intensities.

**Figure 3:**
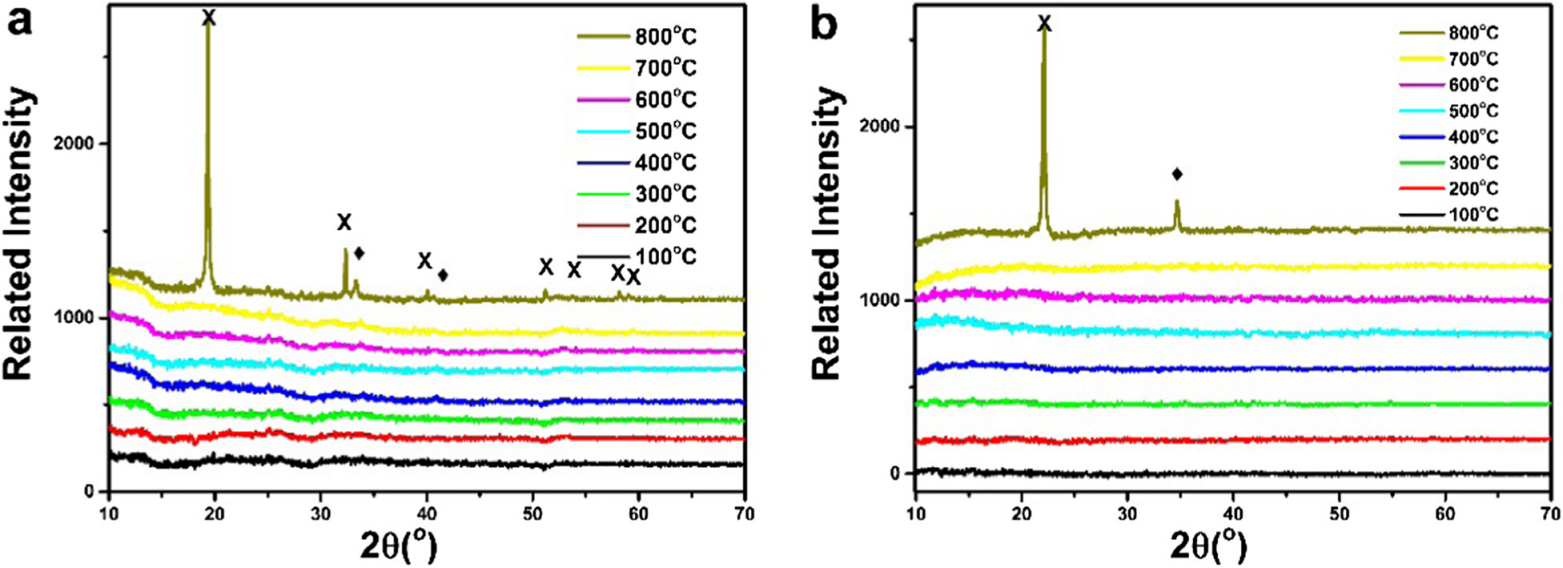
**XRD patterns of Co@SiO**_**2**_ **nanoparticles with temperature (multiplication sign) β-SiO**_**2**_ **, (black diamond) Co**_**3**_ **O**_**4**_ **. (a)** Air condition and **(b)** N_2_ gas protection condition.

In order to invest the structure changes during the heating process, combining *in situ* XRD and EXAFS techniques were performed. Figure 3 shows the results of the *in situ* XRD measurements. Figure 3a,b represents the measurements in air and N_2_ atmosphere, respectively. In addition, the sample in Figure 3b is the mixture of Co@SiO_2_ and BN powders. No diffraction peaks were observed in spite of being in air or N_2_ atmosphere when the temperature was below 800°C, indicating that the Co@SiO_2_ core-shell nanoparticles are maintained amorphously. However, when the temperature is above 800°C, SiO_2_ and Co_3_O_4_ crystals were clearly observed (Figure 3). It is worth noting that the SiO_2_ shells could not protect the Co cores from oxidizing to Co_3_O_4_, which can be demonstrated in the following EXAFS analysis.

To characterize the structure change of Co cores of the nanoparticles, *in situ* EXAFS technique was used to probe the local atomic structures of Co in the Co@SiO_2_ nanoparticles. *In situ* EXAFS spectra of the Co K edge were fitted with the following EXAFS function [25-27]:

$$ \chi (k)={\displaystyle \sum_j}\frac{N_j{S_0}^2{F}_j(k)}{k{R_j}^2}{e}^{-2{k}^2{\sigma_j}^2}{e}^{-2{R}_j/\lambda (k)} \sin \left[2k{R}_j+{\phi_j}^l(k)\right] $$

where *j* refers to the *j*th coordination shell, *Nj* is the coordination number of the *j*th shell, *S*_0_^2^ is the amplitude reduction factor, *F*_*j*_(*k*) is the element-specific backscattering amplitude, *R*_*j*_ is the average distance between the absorbing atom and the backscattering atoms in the *j*th shell, *λ*(*k*) is the mean free-path length of photoelectron, *σ*_*j*_^2^ is the Debye-Waller factor, and *ϕ*_*j*_^*l*^(*k*) is the phase shift experienced by the photoelectron in the scattering process.

**Figure 4:**
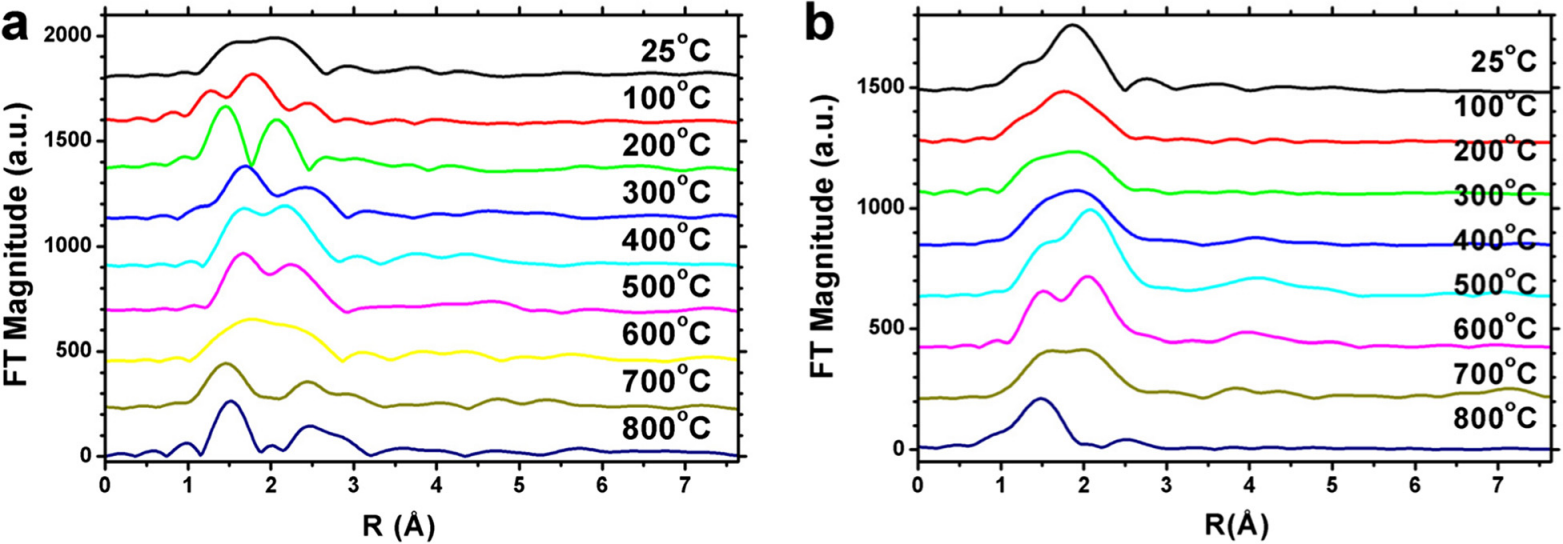
**Phase-uncorrected Fourier transform spectra of Co K-edge EXAFS signals with temperature. (a)** Air condition and **(b)** N_2_ gas protection condition.

**Figure 5:**
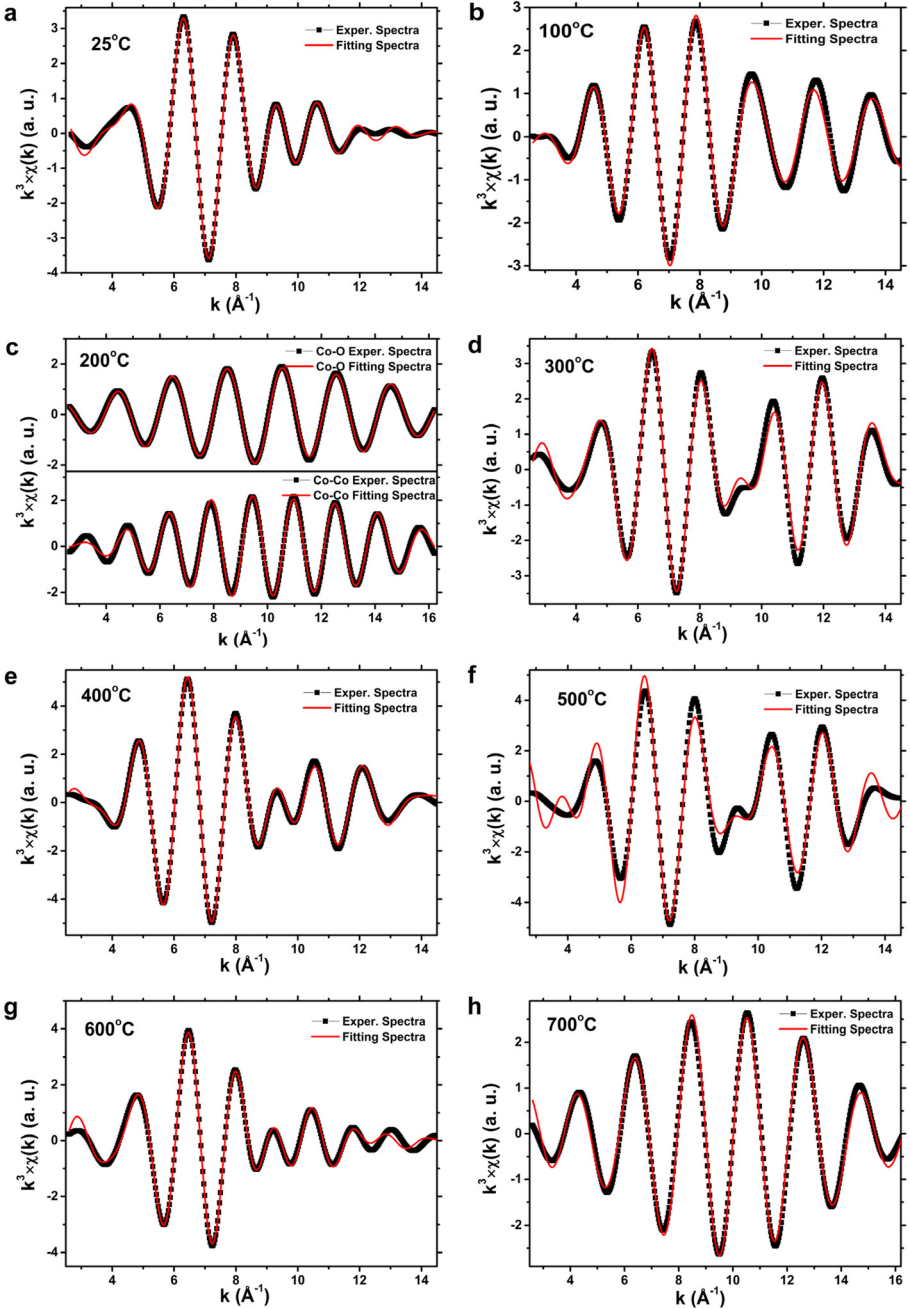
**Fitting results of Co K-edge** ***k*** ^**3**^ **-weighted EXAFS spectra. (a)** to **(h)** figures show the fitting results of Co K edge *k* ^*3*^-weighted EXAFS spectra of Co@SiO_2_ nanoparticles in air condition.

The post-edge background was removed by using a derivative method [28,29]. For the Co@SiO_2_ core-shell nanoparticles in air condition, the Fourier transforms were performed in the *k* range of 2.67 to 14.49 ${\mathring{A}}^{-1}$, and the first Co-Co and Co-O shells were isolated by Fourier filter with *R* range of about 1.10 to 2.70 $\mathring{A}$. Figure 4 shows the Fourier-transformed *k*^3^-weighted EXAFS spectra of Co@SiO_2_ samples in air and N_2_ conditions. The amplitudes and phase shifts of Co-Co and Co-O atom pairs were extracted from theory spectra of CoO which was calculated by FEFF 8.0 [26]. For fitting the EXAFS spectra, we consider the peak around 1.5 $\mathring{\mathrm{A}}$ to Co-O bonds and the peak around 2.4 $\mathring{\mathrm{A}}$ to Co-Co bonds respectively. Therefore, the Co-O and Co-Co scattering paths were used to fit the spectra. The amplitude and phase shift of Co-O atom pair were calculated with FEFF 8.0 code, and the amplitude and phase shift of Co-Co were attracted from Co-foil EXAFS measurement. From the Figure 4a, two peaks were observed during the heating process, and Co-O and Co-Co bonds could fit the spectra very well which were shown in Figure 5. It means that in air condition, the Co core nanoparticles were partially oxidized even at room temperature and then were gradually oxidized to Co_3_O_4_ with the temperature rising to 800°C. However, only one peak was indicated in the N_2_ gas condition when the temperature was below 400°C (Figure 4b). With further increase in temperature, the second peak appeared. Consequently, in N_2_ gas protection condition, the Co core nanoparticles could be oxidized to Co_x_O_y_ when the temperature was above 400°C, and below that temperature, the Co core nanoparticles are dominated by Co^0^ state. Unfortunately, the EXAFS spectra of Co@SiO_2_ nanoparticles could not be fitted well by Co-O and Co-Co scattering paths. Nevertheless, they showed the same trend as in the air condition.

**Figure 6:**
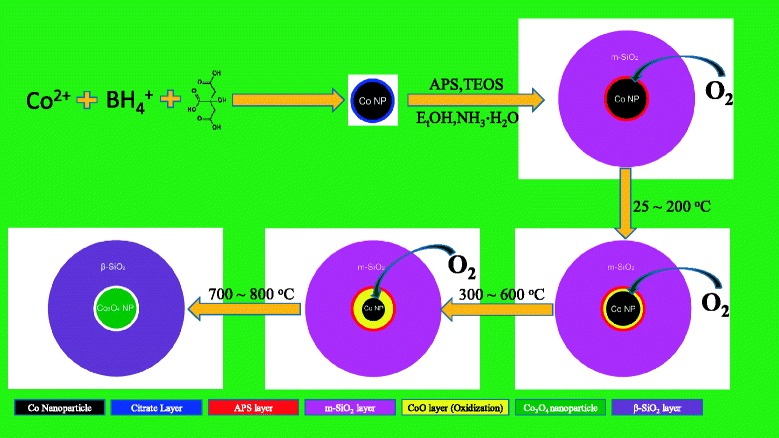
**Schematic illustration of oxidization of Co cores of Co@SiO**_**2**_ **nanoparticles in air condition.**

Comparing the measurements in Figure 4a,b, we can make a conclusion that the Co@SiO_2_ core-shell nanoparticles can be oxidized to Co_3_O_4_, in spite of the protection of SiO_2_ shell. In other words, the SiO_2_ shell cannot protect the Co nanoparticles from being oxidized to Co_3_O_4_, but they could exhibit different behaviors in the air and N_2_ gas conditions. For the nanoparticles in air condition, the O_2_ in air can get onto the Co cores easily because the SiO_2_ shell is in mesoporous state. So even at room temperature, the Co core nanoparticles could be oxidized to CoO which were demonstrated by EXAFS and XANES measurements. In the first step, only the surface atoms of Co cores were oxidized by O_2_. As the temperature increases up to 300°C, the organic ligands leave off the Co core surface, and the Co surface were oxidized to CoO. With further increase in temperature, the CoO layer increased, which was reflected from the *k* space of XAFS spectra (Figure 5), and Figure 5a to h shows the fitting results of Co K edge *k*^*3*^-weighted EXAFS spectra of Co@SiO_2_ nanoparticles in air condition. Finally, the Co core nanoparticles were oxidized thoroughly to Co_3_O_4_ when temperature reaches 800°C. Figure 6 gives the diagrammatic sketch of this procedure.

**Figure 7:**
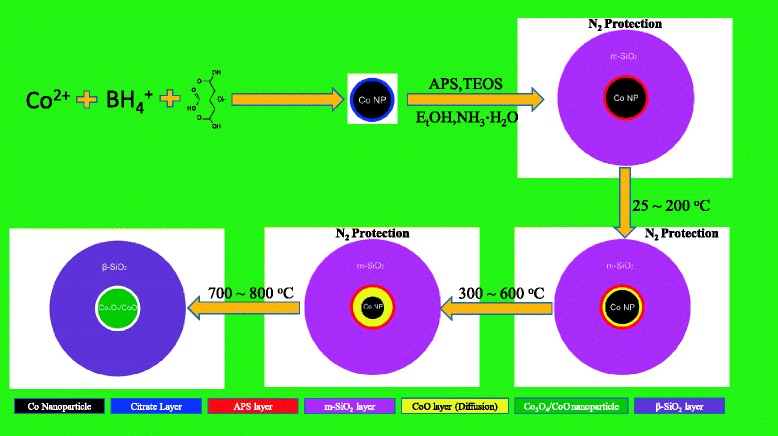
**Schematic illustration of oxidization of Co cores of Co@SiO**_**2**_ **nanoparticles in N**_**2**_ **protection.**

**Figure 8:**
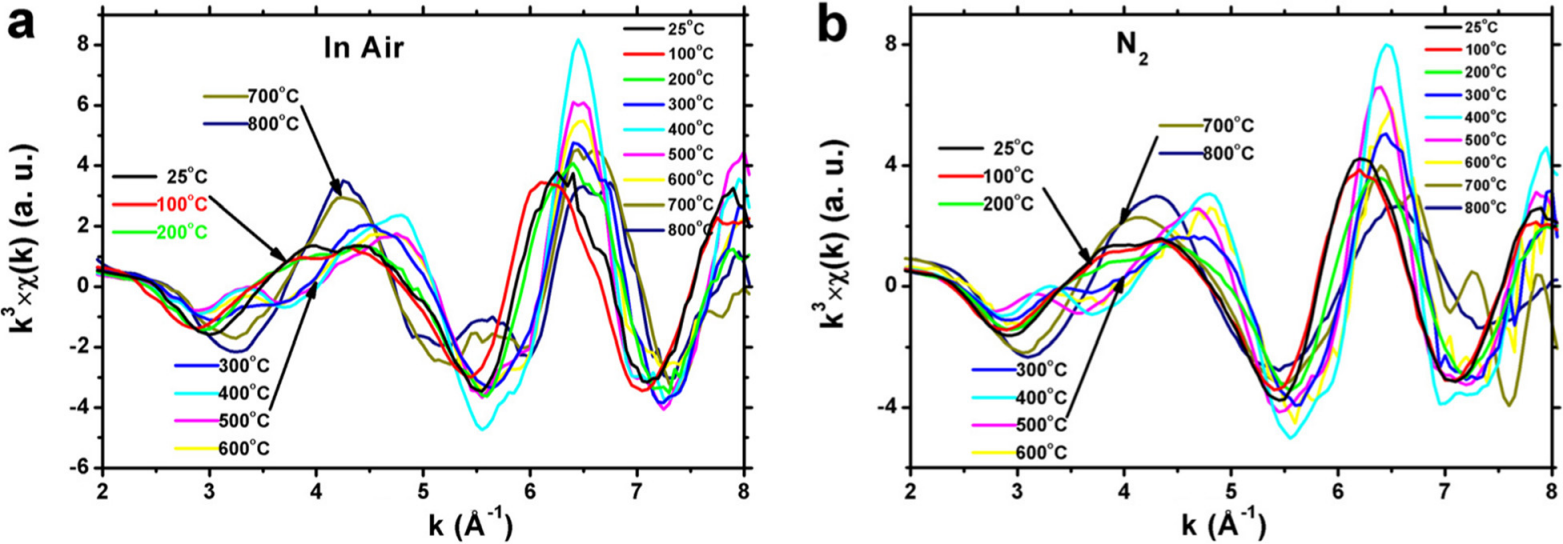
**EXAFS** ***k*** ^**3**^ ***χ*** **(** ***k*** **) function of Co K edge of Co@SiO**_**2**_ **nanoparticles in air (a) and in N**_**2**_ **gas condition (b).**

Figure 7 gives the diagrammatic sketch of the oxidation procedure in the N_2_ gas protection condition. The oxidization of Co core is much different from that in air condition. No exotic O atoms come into the Co@SiO_2_ during the heating process. Thus, the O atoms could only be from the SiO_2_ shells. At low temperature, there is no or seldom Co-O bond existing in the system and the Co-Co bond is dominant. When the temperature was above 300°C, the diffusion effect of O at the Co core surface becomes obvious, and a Co-O band layer will be formed at the interface between Co cores and SiO_2_ shells, which is demonstrated by XAFS in *k* space (cf. Figure 8b). With further increase in temperature, a lot of O atoms in SiO_2_ shell could diffuse into the Co cores and resulting in the increase of the Co-O layer. In the Figure 4b, a peak around 1.5 $\mathring{\mathrm{A}}$ appeared corresponding to the Co-O bond. The m-SiO_2_ shell makes phase transition to β-SiO_2_ around 600°C; it is well known that the O becomes active during the phase transition process, so the diffusion of O into Co core is much faster, and leading further oxidization of the Co core. According to Figures 4b and 8b, the Co nanoparticles are likely oxidized to CoO/Co_3_O_4_ composite because the O and Si are in stoichiometric equal (Si:O = 1:2) in SiO_2_ shell.

For fitting the EXAFS spectra of Co@SiO_2_ in N_2_ gas protection, the signal around 1.5 $\mathring{\mathrm{A}}$ was also considered to be from the CoSi_2_, but no reasonable fitting parameters can be obtained. However, the formation of CoSi_2_ during the heating and annealing process could not be excluded, accounting into the trace amount of which cannot be identified by XAFS technique.

In order to describe the oxidization process precisely, the Co K-edge *k*^3^-weighted Fourier transformed function was studied as shown in Figure 8. We can observe that in the range of $k=3.0\sim 6.0\;{\mathring{A}}^{-1}$, the oxidization procedure can be divided into three steps in spite of being in air and N_2_ gas conditions. From room temperature to 200°C, the Co core is mostly in Co^0^ and may exist some amount of Co^2+^. As the temperature increases to 600°C, the Co core is oxidized to Co^2+^ gradually. When temperature is higher than 800°C, the Co core is transformed into Co_3_O_4_ thoroughly (in air) or partially (in N_2_ gas, CoO/Co_3_O_4_ complex).

## Conclusions

In summary, the Co@SiO_2_ core-shell nanoparticles were prepared, and *in situ* XRD and EXAFS techniques were used to detect the oxidization process of the Co core with temperature increases to 800°C in both air and N_2_ gas conditions. We find that there are three steps during the heating program control temperature procedure in spite of being in air or in N_2_ gas protection. In the first step from room temperature to 200°C, the Co cores are mainly in Co^0^ state as well as some amount of Co^2+^ ions. When temperature is above 300°C, the interface between Co core and SiO_2_ shell is gradually oxidized into Co^2+^, and the CoO layer appears. With temperature increases to 800°C, the Co cores are oxidized to Co_3_O_4_ or Co_3_O_4_/CoO. Nevertheless, the oxidization kinetics of Co cores is strongly influenced by gas condition. In the air condition, the O_2_ in the air could get through easily onto the surface of the Co cores and induces the oxidization of the Co cores due to mesoporous nature of SiO_2_ shells. In the case of N_2_ gas condition, the O atoms could only come from the SiO_2_ shells, so the diffusion effect of O atoms at the interface between Co core and SiO_2_ shell is the main factor. Our current work could provide some hints to study the stability property of core-shell nanoparticles at high temperature.
